# Rare complications of pessary use: A systematic review of case reports

**DOI:** 10.1002/bco2.174

**Published:** 2022-07-05

**Authors:** Stefan Dabic, Christina Sze, Stephanie Sansone, Bilal Chughtai

**Affiliations:** ^1^ Department of Urology Weill Cornell Medicine/New York Presbyterian New York New York USA

**Keywords:** adverse effects, fistula, incontinence, pessary, prolapse, rare complications

## Abstract

**Introduction:**

Pessaries are desirable for its overall safety profiles. Serious complications have been reported; however, there is little summative evidence. This systematic review aimed to consolidate all reported serious outcomes from pessaries usage to better identify and counsel patients who might be at higher risk of developing these adverse events.

**Methods:**

We performed a systematic literature review using search terms such as ‘prolapse’, ‘stress urinary incontinence’ and ‘pessary or pessaries or pessarium’ on PubMed, Embase and CINAHL. A total of 36 articles were identified. Patient‐level data were extracted from case reports to further describe complications on an individual level.

**Results:**

Overall median age of the patients was 82 years (range 62–98). The most frequent complications were vesicovaginal fistula (25%, *n* = 9/36), rectovaginal fistula (19%, *n* = 7/36), vaginal impaction (11%, *n* = 4/36) and vaginal evisceration of small bowel through vaginal vault (8%, *n* = 3/36). In the vesicovaginal fistula cohort, none of the patients had a history of radiation, and two had histories of total abdominal hysterectomy (22%). In the rectovaginal fistula cohort, one patient had a history of pelvic radiation for rectal squamous cell carcinoma, and another had a history of chronic steroid use for rheumatoid arthritis. No other risk factors were reported in the other groups. Ring and Gellhorn were the most represented pessary types among the studies, 16 (44%) and 12 (33%), respectively. No complications were reported with surgical and non‐surgical treatment of the complications.

**Conclusion:**

Pessaries are a reasonable and durable treatment for POP with exceedingly rare reports of severe adverse complications. The ideal candidate for pessary should have a good self‐care index. Studies to determine causative factors of the more serious adverse events are needed; however, this may be difficult given the long follow‐up that is required.

## 1 INTRODUCTION

Pessaries are vaginal support devices that are used for non‐surgical management of pelvic organ prolapse (POP), urinary incontinence and other pelvic floor disorders.^1^, ^2^, ^3^, ^4^ Their use dates to fifth century BCE and has continued to be great options for patients who have not completed childbearing or are poor surgical candidates.^2^, ^5^, ^6^ They also serve as temporary mechanical management for those who elected surgical management but are awaiting surgical optimization.^7^, ^8^, ^9^

With technological advancement, pessaries are often advocated because of their inert compositions and overall safety profiles. The most common side effects include vaginal discharge and odour. Although serious complications such as vesicovaginal fistula, rectovaginal fistula, erosion and subsequent impact have all been reported, there is little summative evidence detailing their prevalence and unique outcomes.^10^ This systematic review aims to consolidate all reported serious outcomes from pessaries usage to better identify and counsel patients who might be at higher risk of developing these adverse events.

## 2 METHODS

### 2.1 Sources

**FIGURE 1:**
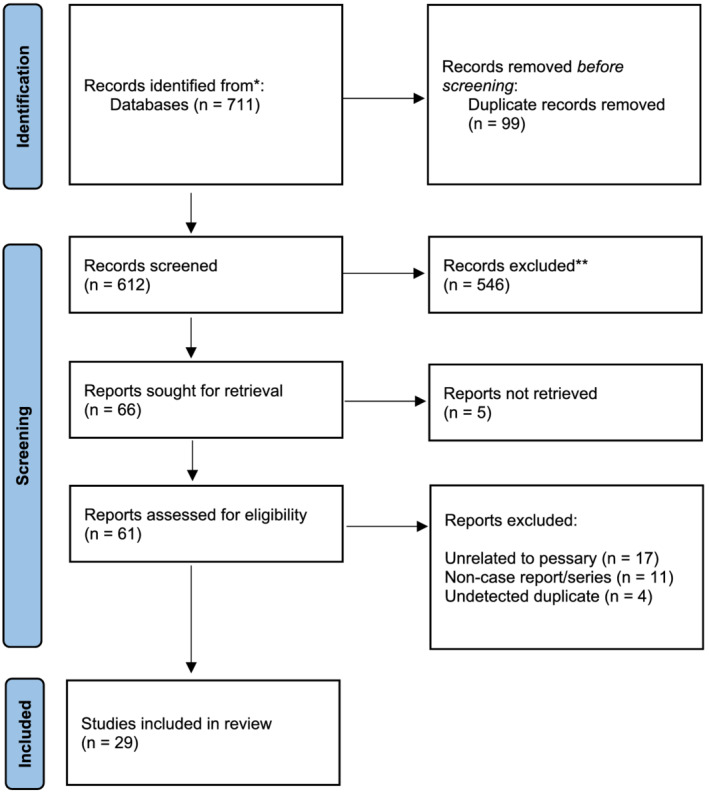
PRISMA flow diagram for study inclusion

We performed a systematic literature review that respected the Preferred Reporting Items for Systematic Reviews and Meta‐Analyses (PRISMA) guidelines (Figure 1).^11^ The review protocol was registered with PROSPERO prior to data extraction (registration no. CRD42020191677).

A search was performed using the search terms such as ‘prolapse’, ‘stress urinary incontinence’ and ‘pessary or pessaries or pessarium’ on PubMed, Embase and CINAHL.

### 2.2 Study selection

After filtering for articles written in the English language, the searches yielded 711 studies (PubMed: 189, Embase: 452, CINAHL: 70). The web‐based review manager Rayyan was used to identify duplicate records and conduct a primary abstract screen (Figure 1).^12^ After deduplication, 612 unique records were screened by two independent reviewers (SD, CS). The inclusion criteria were studies related to complications related to pessary use with the study design of case report or case series. After this first pass, 66 articles remained for primary manuscript screen (SD). Criteria for exclusion included index complication unrelated to pessary use, undetected duplicates and studies that were not case reports or case series study design.

#### 2.2.1 Data extraction

After full‐text screening, 36 studies were selected for data extraction. Data extraction was performed and verified by SD. Variables related to patient age at presentation, duration of pessary use, pessary type, pessary neglect, presenting symptoms, complication, treatment of complication and outcome were all abstracted into a study‐level tabular format.

#### 2.2.2 Risk of bias assessment

Risk of bias assessment was performed for all included studies (Table S1). Because all studies were case reports or case series, the risk of bias assessment performed was the Joanna Briggs Institute Critical Appraisal Checklist.^13^

#### 2.2.3 Descriptive analysis

Patient‐level data were extracted from case series studies to further describe complications on an individual level. Each complication description for each patient was then categorized in order to create a summary of the rare outcomes related to each pessary type.

## 3 RESULTS

### 3.1 Description of study and study population

Thirty‐three of the 36 pessaries (92%) included in the patient‐level analysis were indicated for POP, with two indications not reported and one being for unspecified urinary incontinence. Among these devices, there were 16 ring pessaries (44%), 12 Gellhorn (33%), three shelf pessaries (8%), two mixed pessaries (5%), one cube pessary (3%), ones Gehrung pessary (3%) and one (3%) not reported (Table 1).

**TABLE 1:** Patient‐level abstraction data from case reports

Article	Age	Patient risk factors	Pessary type	Duration of use (years)	Pessary Indication	Pessary management	Complication category	Treatment
Asumpinwong et al. 2019^14^	84	T2DM, CAD	Ring	7	Pelvic organ prolapse	Lost to follow‐up after 1 year of weekly pessary care and routine gyn visits	Uterine migration	Hysterectomy and sacrocolpopexy
Cabral Ribeiro et al. 2017^15^	87	Alzheimer's dementia	Ring	‐	‐	‐	Vaginal impaction	Removal of pessary
Christopher et al. 2017^16^	62	Pelvic radiation (rectal SCC)	Ring	0.17	Pelvic organ prolapse	‐	RVF	Surgical repair of defect
Liu et al. 2017^17^	81	‐	Ring	18	Pelvic organ prolapse	Reported self‐maintenance every 2–3 days^a^	VVF	Surgical repair of defect
Liu et al. 2017^17^	80	Hysterectomy	‐	5	Pelvic organ prolapse	‐^a^	VVF	Surgical repair of defect
Reisenauer et al. 2017^18^	87	‐	Ring	16	Pelvic organ prolapse	Routine monthly gyn visits with most recent placement at 6 months prior	RVF	Ileostomy and delayed surgical repair of defect
Gordon et al. 2015^19^	88	Dementia, HTN, PVD	Gellhorn	‐	Pelvic organ prolapse	Neglect	RVF	Removal of pessary with colostomy
Andrikopoulou et al. 2015^20^	91	Metastatic lymphoma, hysterectomy	Gellhorn	14	Pelvic organ prolapse	Neglect	Vaginal impaction	Pessary removal under general anaesthesia
Abdool et al. 2015^21^	64	‐	Ring	‐	Pelvic organ prolapse	Routine 3‐ to 6‐month gyn visits^a^	Vaginal impaction	Pessary removal
Gordon et al. 2015^19^	64	MI, HTN, T2DM, CAD, PVD, arthritis	Donut + Gellhorn	‐	Pelvic organ prolapse	Routine month gyn visits	RVF	Removal of pessary with bowel diversion
Taillon et al. 2015^22^	74	‐	Gellhorn	1	Pelvic organ prolapse	‐	Uterine migration	Removal and replacement with ring pessary
Penrose et al. 2014^23^	82	‐	Ring	2.5	Pelvic organ prolapse	Routine 6‐month exchanges^a^	VVF	Urinary diversion without repair of VVF
Torbey et al. 2014^24^	75	RA (with daily corticosteroid and weekly methotrexate)	Cube	3	Pelvic organ prolapse	Routine monthly gyn visits with replacement every 4 months	RVF	Removal of pessary with bowel diversion
Cichowski et al. 2013^25^	85	Hysterectomy	Gellhorn	‐	Pelvic organ prolapse	Routine gyn visits	RVF	Removal of pessary with conservative management of fistula
Rogo‐Gupta et al.2012^26^	79	‐	Gellhorn	11	Pelvic organ prolapse	Neglect	VVF	Surgical repair of defect
Siddiqui et al. 2011^27^	79	CVD	Shelf	‐	Pelvic organ prolapse	‐	Vaginal evisceration	Surgical repair of defect
Walker et al. 2011^28^	86	Multiple hospitalization for psychosis	Shelf	13	Pelvic organ prolapse	Neglect	Urethrovaginal fistula	Conservative management
Rubin et al. 2010^29^	82	Breast cancer, hysterectomy	Gellhorn	‐	Pelvic organ prolapse	‐	Vaginal evisceration	Exploratory laparotomy with colpocleisis
Berger et al. 2009^30^	81	‐	Ring	28	Pelvic organ prolapse	Neglect	Vaginal evisceration	Removal of pessary
Esin et al. 2008^31^	85	Deafness, blindness, CHF	Gellhorn	10	Pelvic organ prolapse	Neglect	VVF	Surgical repair of defect and colpocleisis
Esin et al. 2008^31^	93	Hip fracture	Gellhorn	4	Pelvic organ prolapse	‐	VVF	Surgical repair of defect and colpocleisis
Gill et al. 2008^32^	88	‐	Gellhorn	0.01	Pelvic organ prolapse	‐^a^	Uterovaginal strangulation	Hysterectomy
Powers et al. 2008^33^	70	‐	Gellhorn	‐	‐	Neglect	RVF	Removal of pessary transanally
Kaaki et al. 2007^34^	84	‐	Gehrung	12	Pelvic organ prolapse	Routine gyn visits	VVF	Surgical repair of defect and colpocleisis
Kim et al. 2005^35^	80	Hysterectomy, bilateral TKA, RA	Gellhorn	2	Pelvic organ prolapse	Routine gyn visits	VVF	Surgical repair of defect and colpocleisis
Tatar et al. 2005^36^	94	‐	Ring	0.67	Urinary incontinence	‐	Continuous leakage	Removal of pessary
Ka Yu et al. 2004^37^	64	Marfan syndrome	Ring	7	Pelvic organ prolapse	Routine gyn visits	Cervical incarceration	Surgical removal of pessary with plans to perform cervical amputation later
Wheeler et al. 2004^38^	75	CVD	Shelf	3	Pelvic organ prolapse	Routine gyn visits	Sepsis	Exploratory laparotomy with removal of pessary
Sasso et al. 2003^39^	67	HTN, RA	Mixed	7	Pelvic organ prolapse	Routine gyn visits^a^	Vaginal ulceration	Removal and replacement of pessary
Chou et al. 2003^40^	82	‐	Ring	10	Pelvic organ prolapse	Neglect	Vaginal impaction	Surgical removal of pessary
Grody et al. 1999^41^	98	‐	Gellhorn	18	Pelvic organ prolapse	Neglect	VVF	Surgical repair of defect and colpocleisis
Roberge et al. 1999^42^	70	T2DM, COPD	Ring	0.33	Pelvic organ prolapse	Neglect	Sepsis	Surgical removal of pessary
Roberge et al. 1999^42^	85	HTN, CAD, dementia	Ring	‐	Pelvic organ prolapse	Neglect	Sepsis	Surgical removal of pessary
Sivasuriya et al. 1987^43^	80	‐	Ring	10	Pelvic organ prolapse	Routine monthly gyn visits	Cervical incarceration	Surgical removal of pessary
Binnie et al. 1964^44^	66	‐	Ring	‐	Pelvic organ prolapse	Routine gyn visits	Uterine strangulation	Surgical removal of pessary

Abbreviations: CAD, coronary artery disease; CHF, congestive heart failure; COPD, chronic obstructive pulmonary disease; GYN, gynaecologic; HTN, hypertension; MI, myocardial infarction; OSA, obstructive sleep apnoea; PVD, peripheral vascular disease; RA, rheumatoid arthritis; RVF, rectovaginal fistula; T2DM, Type 2 diabetes mellitus; TKA, total knee arthroplasty; VVF, vesicovaginal fistula.

^a^ Vaginal oestrogen cream use.

Median duration of pessary use before the presenting complication was not calculated due to a large proportion of missing data. These complication time points ranged from the initial pessary fitting to 28 years following placement. The most common complication was vesicovaginal fistula formation, which was observed in nine of the 36 patients (25%) represented in the case reports. The next most frequent complications were rectovaginal fistula (19%, *n* = 7/36), vaginal impaction (11%, *n* = 4/36) and vaginal evisceration of small bowel through vaginal vault (8%, *n* = 3/36). The remaining complications were observed in only one or two case reports.

The median age of patients who experienced vesicovaginal fistula was 82 years (range 79–98), with urinary incontinence as the presenting symptom in 89% of these patients (*n* = 8/9) and 33% reporting bleeding or haematuria (*n* = 3/9) (Table S2). Median duration of pessary use was 10 years (range: 6 months to 18 years). None of the patients had a history of radiation, and two had histories of total abdominal hysterectomy (22%). In this cohort, the most common type of pessary used was a Gellhorn (44%, *n* = 5/9), followed by ring (22%, *n* = 2/9). Eight patients (89%) were treated with surgical repair of the fistula, while one patient (11%) was treated with urostomy with ileal conduit. Of the patients who received surgical repair, five (56%) underwent partial or complete colpocleisis as well. One complication was reported with urostomy with ileal conduit in which the patient experienced superficial wound dehiscence. All other patients reported no complications, with reported full continence at various follow‐up times.

For the seven patients with rectovaginal fistula, the median patient age was 75 years, ranging from 62 to 88 (Table S2). All pessary types were represented in this group with no type having a majority. The most common presenting symptom was stool leakage through the vagina in three of the seven patients (43%). Of note, one of these patients had a history of pelvic radiation for rectal squamous cell carcinoma, another had a history of chronic steroid use for rheumatoid arthritis, and the third did not have a reported medical history. Four patients (57%, *n* = 4/7) were treated with removal of pessary and colostomy, one was treated with fistula coverage with left inferior rectus (14%, *n* = 1/7), and two were treated with only removal of pessary (29%, *n* = 2/7). Most patients (71%, *n* = 5/7) had successful resolution of rectovaginal fistula at various follow‐up times, whereas one was lost to follow‐up, and another had no resolution.

For patients with vaginal incarceration, the median age was 73 (64–91) with three of the four pessaries being rings and one being a Gellhorn (Table S4). One 91‐year‐old patient with an extensive complicated medical history had used the pessary for 14 years. Another patient had no reported duration of use but did have a history of Alzheimer's dementia. The most common presenting symptoms in this cohort were bleeding and pain in two of the patients. Pessary removal with or without local excision of granulation tissue was performed in all four patients. One patient required surgical removal, one had removal while under general anaesthesia, and another had removal under local anaesthesia. All four patients had resolution of symptoms after removal of pessary.

Table S4 outlines the complications by pessary type. A fistulous complication was observed in each pessary type. Ring and Gellhorn were the most represented pessary types among the studies, 16 (44%) and 12 (33%) total patients, respectively. Of note, six (50%) of the 12 Gellhorn pessaries and four (25%) of the 16 ring pessaries were reported to be neglected by the patient.

## 4 DISCUSSION

Despite the severity of symptoms and complications related to pessary use, most patients included in this systematic review of case reports had resolution of symptoms following judicious surgical and non‐surgical treatment of the complications. These complications did not necessarily preclude treatment of their index POP. Ring and Gellhorn pessaries made up the majority (83%) of all pessary types; however, our results do not suggest association with any one specific adverse outcome.

Our cohort had a median age of 82 years (range 62–98), suggesting that advanced age may contribute to the development rare outcomes; however, evidence seems to support higher utilization of surgery in this population to avoid the higher discontinuation and complication rates. In a large retrospective study consisting of 304 women, vaginal erosions were three times more likely to occur in advanced age defined as >75 years (HR 3.2, 95% CI 1.6–6.3).^45^ Additionally, patients in the advanced age category had a higher percentage of discontinuation rate than the younger population (87.5% vs 80.8% at 1 year and 62.1% vs 37.8% at 5 years). Ultimately, 25% of patients who successfully completed a pessary trial chose surgical repair, and 17% left their prolapse untreated. Additionally, closer follow‐up does not necessarily lead to better outcomes. In a double‐blinded, randomized controlled trial, the overall complication rate in the group with 6‐month interval follow‐up was higher than in the group with 3‐month interval follow‐up; however, this was not statistically significant.^46^ Also, there was no significant difference between the groups in patient satisfaction scores or prolapse‐related symptoms.^46^ Pessaries are desirable because they are often thought of as viable options for elderly patients who are not good surgical candidates due to comorbidities such as advanced age. However, robotic pelvic surgery in the elderly population is feasible in the hands of experienced surgeons.^47^ There should be a lower threshold for referral for surgical management before providers categorize a patient as truly non‐operative. Additionally, there were no complications reported after surgical treatment, implying these patients were able tolerate some sort of surgery.

Although rare complications are often attributed to neglect, there are no comparative studies that formally demonstrate this. In our cohort, 11/36 (30%) of complications were reported to be attributed to neglect, which suggested that definitive treatment is more desirable in this population. In our cohort, pessary neglect was observed in some of the patients who had vesicovaginal (22%) or rectovaginal fistula (28.6%). Many of the studies (42%) reported that patients had reliable and consistent gynaecologic follow‐up; however, recall bias limited the validity of their reported pessary care. Besides poor surgical candidacy, a better qualification for pessary management of POP should be the ability to self‐care. Pessary maintenance requires diligence. The Society of Obstetricians and Gynaecologists of Canada suggest that women who can remove and clean the pessary with soap and water can do so weekly.^4^ In a 5‐year prospective study of 249 patients, Ma et al. demonstrated that the incapability of self‐care (OR = 2.6 95% CI 1.3–5.1) was a risk factor for pessary discontinuation.^48^ Roughly 11% of patients in our cohort were either in long‐term care facilities or under the care of home nurse or close family members—therefore lacked self‐care. Depending on the regulations at the care institution and caretaker's comfort level, pessary maintenance may be further overlooked.^49^ Therefore, observation can also be offered as a reasonable option for these patients with POP. Gilchrist et al. demonstrated that in a cohort of 64 patients who elected observation as primary management of symptomatic POP with median follow‐up of 16 months, prolapse progression (defined as >2 cm increase in leading edge) was seen in 19% of patients. The majority (63%) of patients elected continued observation, and those that elected intervention either with pessary or surgical correction had no greater worsening of prolapse on exam, suggesting that treatment compliance requires communication and validation from physician as well as setting realistic expectations on outcomes.^50^ Surgical or observational approaches may be more reasonable options for patients with advanced age and poor self‐care index.

Risk factors compromising tissue integrity such histories of radiation and steroid were not strongly represented. External radiation therapy and steroid use were only reported in a minority of patients, with only one (3%) report of pelvic radiation, two (5%) reports of corticosteroid use and two patients with RA. Additionally, only five (13.9%) of the patients listed in our analysis reported patient use of vaginal oestrogen cream with their pessary, which is often used to strengthen vaginal epithelium in postmenopausal women. Some studies suggested that oestrogen cream itself does not appear to be associated with decreased risk of vaginal erosion, though it is associated with decreased discontinuation of pessary.^51^ In a randomized control trial of 40 postmenopausal women, 6 weeks of local oestrogen cream did not affect vaginal health in pessary use with POP.^52^ Additionally, local oestrogen cream did not change the difficulty to insert and remove the pessary.^14^ Therefore history of radiation or abdominal surgeries should not preclude pessary use.

Our review is not without limitations. Because this review relies on the publication and report of adverse events, it does not capture the true prevalence and may under‐report these complications. Therefore, our review is intended to provide a systematic collection of reported outcomes as reference for those that may encounter these rare complications. These case reports are variable in the information they provide; therefore, limited correlation and causality can be made. Additionally, our review does encompass all the possible rare complications associated with pessaries. Conversely, they may also exaggerate these complications that may no longer be relevant with technological advancement, guideline changes and diligent practice patterns.

## 5 CONCLUSION

Serious complications associated with vaginal pessary use are exceedingly rare, with only 36 serious adverse events reported from 1964 to 2019. Along with comorbidities such as advanced age, providers should consider a patient's self‐care index when recommending pessary as management. Studies to determine causative factors of the more serious adverse events are needed but may be difficult given long follow‐up is required.

## DISCLOSURE OF INTERESTS

This work is supported in part by Cosm Medical.

## AUTHOR CONTRIBUTIONS

Study concept and design: Bilal Chughtai. Acquisition of data: Stefan Dabic, Christina Sze, Stephanie Sansone. Drafting of the manuscript: Stefan Dabic, Christina Sze. Critical revision of the manuscript for important intellectual content: Stefan Dabic, Christina Sze, Stephanie Sansone, Bilal Chughtai.

## Supporting information


**Table S1:** Case reports with complications of vesicovaginal fistula
**Table S2:** Case reports with complications of rectovaginal fistula
**Table S3:** Case reports with complications of vaginal Impaction
**Table S4:** Complications observed by pessary type
