# Supplementary material for: Rare complications of pessary use: A systematic review of case reports
Source: BJUI Compass. 2022 Jul 5;3(6):415–23. doi: 10.1002/bco2.174 (PMC9579882; doi:10.1002/bco2.174)
Supplement: Supplementary file 1 — Table S1: Case reports with complications of vesicovaginal fistula Table S2: Case reports with complications of rectovaginal fistula Table S3: Case reports with complications of vaginal Impaction Table S4: Complications observed by pessary type [file BCO2-3-415-s001.docx]

**Supplemental**

**Table 1:** Case reports with complications of vesicovaginal fistula

| Article | Age | Risk Factors | Pessary type | Duration of use (years) | Presenting symptoms | Treatment | Outcome |
| --- | --- | --- | --- | --- | --- | --- | --- |
| Rogo-Gupta *et al.* 2012^34^ | 79 | - | Gellhorn | 11 | Hematuria  UTI Urinary incontinence  Vaginal bleeding | Surgical repair of defect | No complications reported |
| Liu *et al.* 2017^25^ | 81 | - | Ring | 18 | Urinary incontinence | Surgical repair of defect | No complications reported |
| Liu *et al.* 2017^25^ | 80 | Hysterectomy | - | 5 | Cystocele  Urinary incontinence  Rectocele  Vault prolapse | Surgical repair of defect | No complications reported |
| Penrose *et al.* 2014^31^ | 82 | - | Ring | 2.5 | Persistent vaginal bleeding | Urinary diversion | Superficial wound dehiscence at day 10. VVF unchanged 5 months post-op, with uterus elevated behind stenosed upper vagina. Patient remained dry and managed urostomy independently. |
| Kim *et al.* 2005^44^ | 80 | Hysterectomy | Gellhorn | 2 | Vaginal discomfort | Surgical repair of defect and Colpocleisis | No complications reported |
| Grody *et al.* 1999^50^ | 98 | - | Gellhorn | 18 | Urinary incontinence | Surgical repair of defect and Colpocleisis | No complications reported |
| Esin *et al.* 2008^40^ | 85 | Deafness, blindness | Gellhorn | 10 | Extreme pain,  Urinary incontinence | Surgical repair of defect and Colpocleisis | No complications reported |
| Esin *et al.* 2008^40^ | 93 | - | Gellhorn | 4 | Urinary incontinence | Surgical repair of defect and Colpocleisis | Residual small VVF after repair 6 weeks post op, resolved after 4 weeks suprapubic catheter.  Remained continent |
| Kaaki *et al.* 2007^43^ | 84 | - | Gehrung | 12 | Urinary incontinence | Surgical repair of defect and Colpocleisis | No complications reported |
| UTI: urinary tract infection | | | | | | | |

**Table 2:** Case reports with complications of rectovaginal fistula

| Articles | Age | Risk Factors | Pessary type | Duration of use (years) | Presenting symptoms | Treatment | Outcome |
| --- | --- | --- | --- | --- | --- | --- | --- |
| Powers *et al.*2008^42^ | 70 | - | Gellhorn | - | Extreme pain | Removal of pessary transanally | Lost to follow-up |
| Torbey *et al.* 2014^32^ | 75 | RA (with daily corticosteroid and weekly methotrexate) | Cube | 3 | Vaginal pain  Vaginal discharge | Removal of pessary with fecal diversion (colostomy) | No longer has feculent discharge. Fistula status was not reported. |
| Gordon *et al.* 2015^27^ | 88 | Dementia, HTN, PVD | Gellhorn | - | Abdominal pain | Removal of pessary with fecal diversion (colostomy) | Fistula healed on follow-up visit |
| Gordon *et al.* 2015^27^ | 64 | MI, HTN, T2DM, CAD, PVD, dyslipidemia | Donut + Gellhorn | - | Pain  incontinence | Removal of pessary with fecal diversion (colostomy) | Fistula did not heal completely even after 3 months. Hysterectomy and colpopexy was performed for prolapse. |
| Cichowski *et al.*2013^33^ | 85 | Hysterectomy | Gellhorn | - | Vaginal discomfort | Removal of pessary with conservative management of fistula | Fistula healed on follow-up visit |
| Reisenauer *et al.*2017^26^ | 87 | - | Ring | 16 | Vaginal discharge | Removal of pessary with fecal diversion (colostomy) and delayed surgical repair | Fistula healed on follow-up visit |
| Christopher *et al.*2017^24^ | 62 | Pelvic radiation (rectal SCC) | Ring | 0.17 | Vaginal discharge | Surgical repair of defect with flap | Post-surgical course complicated by superior mesenteric artery syndrome however recovered after 12-day hospital stay |

**Table 3:** Case reports with complications of vaginal Impaction

| Articles | Age | Risk Factors | Pessary type | Duration of use (years) | Presenting symptoms | Treatment | Outcome |
| --- | --- | --- | --- | --- | --- | --- | --- |
| Andrikopoulou *et al.* 2015^28^ | 91 | Hysterectomy | Gellhorn | 14 | Vaginal pain | Pessary removal under general anesthesia | Uneventful recovery with discharge 1 week post-removal. Asymptomatic anterior/apical prolapse at 3 months post-operative. |
| Abdool *et al.* 2015^29^ | 64 | - | Ring | - | Inability to remove pessary at routine follow up | Granulation tissue incised under local anesthesia + vaginal cream post op | Good outcome after 6 weeks with request of pessary reinsertion. |
| Chou *et al.* 2003^49^ | 82 | - | Ring | 10 | Urinary incontinence | Surgical removal of pessary | Lower urinary tract symptoms recovered after surgical removal of pessary. |
| Cabral Ribeiro *et al.* 2017^23^ | 87 | Alzheimer's dementia | Ring | - | Intermittent hematuria  Vaginal bleeding | Removal of pessary | Extensive vaginal bleeding after removal but eventually became well. |

**Table 4:** Complications observed by pessary type

| Pessary Type | Indication | Complication | Reference |
| --- | --- | --- | --- |
| Cube | Pelvic organ prolapse | Rectovaginal fistula | Torbey *et al.* 2014 |
| Gehrung | Pelvic organ prolapse | Vesicovaginal fistula | Kaaki *et al.* 2007 |
| Gellhorn | Pelvic organ prolapse | Rectovaginal fistula  Uterine migration  Uterovaginal strangulation  Vaginal evisceration  Vaginal impaction  Vesicovaginal fistula | Gordon *et al.* 2015  Cichowski *et al.* 2013  Rubin *et al.* 2010  Andrikopoulou *et al.* 2015  Rogo-Gupta *et al.*2012  Gill *et al.* 2008  Kim *et al.* 2005  Grody *et al.* 1999  Taillon *et al.* 2015  Esin *et al.* 2008 |
| Mixed | Pelvic organ prolapse | Vaginal ulceration  Rectovaginal fistula | Gordon *et al.* 2015  Sasso *et al.* 2003 |
| Unspecified | Pelvic organ prolapse | Vesicovaginal fistula | Liu *et al.* 2017 |
| Ring | Pelvic organ prolapse  Stress urinary incontinence | Cervical incarceration  Pseudodiarrhea  Rectovaginal fistula  Sepsis  Vaginal cancer  Vaginal evisceration  Vaginal impaction  Vesicovaginal fistula  Uterine migration  Uterine strangulation | Ka Yu *et al.* 2004  Tatar *et al.* 2005  Sivasuriya *et al.* 1987  Reisenauer *et al.* 2017  Christopher *et al.* 2017  Berger *et al.* 2009  Abdool *et al.* 2015  Chou *et al.* 2003  Asumpinwong *et al.* 2019  Cabral Ribeiro *et al.* 2017  Liu *et al.* 2017  Penrose *et al.* 2014  Binnie *et al.* 1964  Roberge *et al.* 1999  Yoshikawa *et al.* 2011 |
| Shelf | Pelvic organ prolapse,  Stress urinary incontinence | Sepsis  Urethrovaginal fistula  Vaginal evisceration | Wheeler *et al.* 2004  Siddiqui *et al.* 2011  Walker *et al.* 2011 |
